# Ubiquitin ligases HUWE1 and NEDD4 cooperatively control signal-dependent PRC2-Ezh1α/β-mediated adaptive stress response pathway in skeletal muscle cells

**DOI:** 10.1186/s13072-019-0322-5

**Published:** 2019-12-19

**Authors:** Peng Liu, Muhammad Shuaib, Huoming Zhang, Seba Nadeef, Valerio Orlando

**Affiliations:** 1 BESE Division, KAUST Environmental Epigenetics Program, King Abdullah University Science and Technology (KAUST), Thuwal, 23955-6900 Saudi Arabia; 2 Core Labs, King Abdullah University of Science and Technology, Thuwal, 23955-6900 Saudi Arabia

**Keywords:** HUWE1, NEDD4, Polycomb, Ubiquitination

## Abstract

**Background:**

While the role of Polycomb group protein-mediated “cell memory” is well established in developmental contexts, little is known about their role in adult tissues and in particular in post-mitotic cells. Emerging evidence assigns a pivotal role in cell plasticity and adaptation. PRC2-Ezh1α/β signaling pathway from cytoplasm to chromatin protects skeletal muscle cells from oxidative stress. However, detailed mechanisms controlling degradation of cytoplasmic Ezh1β and assembly of canonical PRC2-Ezh1α repressive complex remain to be clarified.

**Results:**

Here, we report NEDD4 ubiquitin E3 ligase, as key regulator of Ezh1β. In addition, we report that ubiquitination and degradation of Ezh1β is controlled by another layer of regulation, that is, one specific phosphorylation of serine 560 located at Ezh1β-specific C terminal. Finally, we demonstrate that also Ezh1α needs to be stabilized under stress condition and this stabilization process requires decreased association pattern between another E3 ubiquitin ligase HUWE1.

**Conclusions:**

Together, these results shed light on key components that regulate PRC2-Ezh1α/β pathway to direct modulation of epigenome plasticity and transcriptional output in skeletal muscle cells.

## Background

Besides its role in developmental memory, in adult post-mitotic cells epigenome structure does not appear to be a rigid platform, but rather a dynamic system allowing plasticity to adapt transcriptional programs to naturally changing environmental cues.

Polycomb group proteins (PcG) and the role of H3K27me3 modification in maintaining cellular memory is well known [1–4]. Most of these studies focused on the contribution of Ezh2 histone methyltransferase (HMT) mediating H3K27 methylation. However, mammalian cells contain a second potential H3K27 HMT, Ezh1, highly related to Ezh2. Interestingly, Ezh1 is expressed mostly in embryonic stem cells, in combination with Ezh2, and in adult post-mitotic tissues where Ezh2 is absent [5–7]. In pluripotent cells, Ezh1 could compensate for Ezh2 role in mediating H3K27 methylation both in vitro and in vivo [5, 6, 8, 9]. However, biochemical studies indicate Ezh1 as a weak HMT and its role in post-mitotic cells appears to be complex. Indeed, genome wide studies in skeletal muscle cells provided clues about direct association of Ezh1 with active promoters overlapping with H3K4me3-enriched regions [7, 10, 11] (not H3K27m3) and required for RNA Pol II elongation [10]. However, we previously reported a novel molecular mechanism in skeletal muscle tissue physiology showing the role of PRC2-Ezh1 in modulating H3K27me3 epigenome plasticity in response to oxidative stress [12]. In detail, we found that Ezh1 produced two different isoforms, Ezh1α and Ezh1β exhibiting nucleus and cytosol exclusive localization, respectively. Under oxidative stress or atrophic conditions, Ezh1β is degraded through 26S proteasome system. In this way, EED will escape sequestration by Ezh1β and shuttle from cytosol to nucleus to give rise to canonical PRC2-Ezh1 repressive complex through the interaction with Ezh1α and SUZ12. This triggers Ezh1-dependent H3K27me3 signature and gene silencing at whole genome scale, allowing post-mitotic cells to adapt to oxidative stress thus, unveiling the plastic nature of PRC2-Ezh1 regulation. These findings suggest that Ezh1 is involved in different aspects of transcriptional regulation, both activation and repression, through canonical PRC2-Ezh1 or non-canonical PRC2-Ezh1 pathways. This duality suggests a complex regulated activity of Ezh1 function.

Stoichiometric regulation of PRC2 components is an essential feature of PcG physiology [2]. In different contexts, the role of post-translation modifications (PTMs) in regulating Ezh2 stability, intracellular dynamics and activity has been reported. Smurf2-mediated K421 ubiquitination of Ezh2 and degradation of Ezh2 facilitates hMSC neuron differentiation [13]. Recent data described Praja1 Ubiquitin ligase regulates stability of Ezh2 in a p38 signaling-dependent manner during skeletal myogenesis [14, 15]. Many different ubiquitin E3 ligases have been reported to control Ezh2 in different tumor types [16–18]. These include Trcp1/FBXW1, FBXW7 and TRAF6 [16, 18, 19]. Ubiquitination and degradation of Ezh2 were modulated in a phosphorylation-dependent manner [15, 18, 19]. These findings clearly highlight how different PTMs work cooperatively to regulate activity of Ezh2 under normal differentiation process or tumor cell types.

The mechanisms and role of PTMs in PRC2-Ezh1 dynamics in adult tissues allowing epigenetic adaptive stress response are not known. Here, we report about the ubiquitin ligase dependent signaling dynamics of PRC2-Ezhα/β pathway in skeletal muscle cells. We show that NEDD4 is the major ubiquitin E3 ligase to mediate Ezh1β ubiquitination and degradation. Further, we identify Serine 560 phosphorylation as the signal essential for Ezh1β Ub-E3-ligase dependent degradation. More surprisingly, Ezh1α also exhibited a degradation dynamics in response to stress. We show that Ezh1α requires stabilization under oxidative stress condition to facilitate canonical PRC2-Ezh1α efficient assembly and this function is controlled by HUWE1 ubiquitin E3 ligase. Overall, our data identify NEDD4 and HUWE1, as the key players working in cooperation to regulate the stability of Ezh1β and Ezh1α, respectively, allowing PcG-dependent epigenetic adaptive response in skeletal muscles.

## Results

### Identification of ubiquitin E3 ligases associated with Ezh1β under oxidative stress condition

Our previous work demonstrated that, in response to oxidative stress, Ezh1β would undergo increased ubiquitination and degradation by 26S proteasome pathway [12].

To identity candidate E3 ligases associated with Ezh1β, we used tandem affinity purification (TAP) strategy [20]. We constructed C2C12 cell line constitutively expressing Ezh1β tagged with FLAG and HA at its C terminal. Sub-localization of fusion protein Ezh1β-FLAG-HA was determined in myoblasts and differentiated myotubes. Immunostaining experiments clearly showed that Ezh1β-FLAG-HA localized within cytosol, which is consistent with endogenous Ezh1β localization pattern as previously reported [12] (Additional file 1: Fig. S1a). We also checked relative expression level of Ezh1β-FLAG-HA compared with the endogenous Ezh1β protein. Expression level of exogenous Ezh1β-FLAG-HA was similar to endogenous Ezh1β level (Additional file 1: Fig. S1b). We noticed that in addition to one specific Ezh1β-FH band as predicted molecular weight, another two high molecular bands were detected. To verify whether these two bands were containing Ezh1β polypeptide, we cut these two bands and sent them for mass spectrometry (MS) analysis. Ezh1β was highly represented with highest score in our MS analysis list (Additional file 2: Datasheet 1), confirming that these two bands are also Ezh1β-FH specific, although the reason remains to be elucidated. Further, in vivo ubiquitination assay was performed using Ezh1β-FLAG-HA stable cell line. The results clearly show poly-ubiquitination of Ezh1β levels increased upon oxidative stress condition and exhibit as typical smear profile (Additional file 1: Fig. S2).

**Fig. 1:**
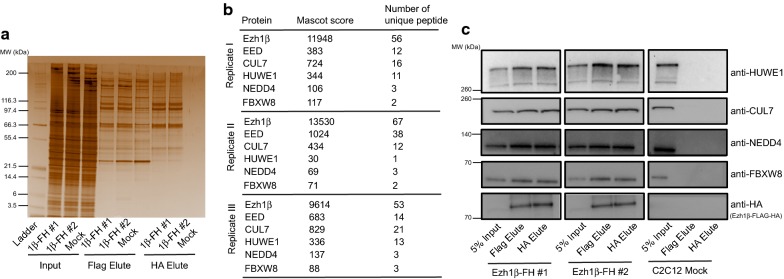
Immunoprecipitation of Ezh1β-associated protein partners under oxidative stress condition. **a** Ezh1β-FH-associated protein complexes were tandemly affinity purified from cytosolic extracts of C2C12 stable cell line which expresses C-terminally FLAG-HA tagged Ezh1β. Flag and HA elutes indicate samples eluted with FLAG and HA peptides, respectively. Flag and HA elute samples were separated by SDS-PAGE and silver stained. Cytosolic protein extracts from C2C12 wild-type cell line were used as mock. Ezh1β-FH expressing C2C12 and normal C2C12 myotube samples, after changing differentiation medium for 2 days, were challenged with 100 μM H_2_O_2_ for 24 h. **b** Tandem affinity enriched Ezh1β-FLAG-HA associated proteins were identified by MS analysis. For each annotated protein, number of unique peptides and MASCOT score were listed. Three independent biological replicates data are presented. **c** Interaction among Ezh1β and ubiquitin E3 ligase: HUWE1, NEDD4 and FBXW8 were validated through co-immunoprecipitation (Co-IP) assay. Ezh1β-FH was immunoprecipitated from cytosolic extracts of two independent C2C12 stable cell line expressing Ezh1β-FH (#1 and #2). Wild-type C2C12 cell line was used as mock control. Ezh1β-FH expressing C2C12 and normal C2C12 myotube samples, after changing differentiation medium for 2 days, were challenged with 100 μM H_2_O_2_ for 24 h. Immunoblot analysis were performed with anti-HUWE1, anti-NEDD4, anti-CUL7, anti-FBXW8 and anti-HA

Following H_2_O_2_ treatment, all HA elute samples were subjected to SDS-PAGE and silver staining (Fig. 1a). In comparison with C2C12 wild-type mock samples, many specific protein partners were immunoprecipitated through TAP assay (Fig. 1a). Then, HA elutes from both Ezh1β-FH stable cell line and wild-type C2C12 cell line were sent for mass spectrometry (MS) analysis and immunoprecipitated interacting protein partners were listed (Additional file 3: Datasheet 2). Specificity was confirmed by EED resulting as top interacting partner of Ezh1β in three independent experiments (Additional file 3: Datasheet 2 and Fig. 1b). Ubiquitin E3 ligases, HUWE1, NEDD4 and CUL7/FBXW8 were identified through this TAP strategy (Additional file 3: Datasheet 2 and Fig. 1b). The specificity of these interactions was further validated through co-immunoprecipitation coupled to western blotting with antibodies specific for those three E3 ligases (Fig. 1c). We conclude that ubiquitin E3 ligase, HUWE1, NEDD4 and FBXW8, are associated with Ezh1β within cytosolic compartment when post-mitotic muscle cells are under oxidative stress conditions.

### NEDD4 is the major ubiquitin E3 ligase mediating Ezh1β ubiquitination and stability upon oxidative stress condition

**Fig. 2:**
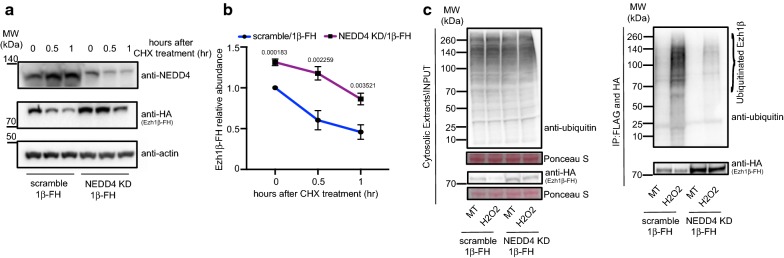
Degradation and poly-ubiquitination of Ezh1β are mainly contributed by NEDD4. **a** CHX chasing assay was performed in scramble/Ezh1β-FH and NEDD4 KD/Ezh1β-FH cell lines stressed with H_2_O_2_. scramble/Ezh1β-FH and NEDD4 KD/Ezh1β-FH cell lines were treated with 100 μM H_2_O_2_ for 24 h. During last hour of H_2_O_2_ treatment, 100 μg/ml cycloheximide (CHX) was added at indicated time points. Total proteins were extracted and immunoblot analysis was performed using anti-NEDD4 and anti-HA, anti-actin was used as loading control. **b** Quantification of remaining Ezh1β-FH protein percentage in **a**. Relative Ezh1β-FH was quantified in comparison remaining Ezh1β-FH with initial total protein amount at indicated CHX treatment time points. Data were expressed as mean ± SD from three biological replicates. ImageJ software was used to determine protein abundance. Values above each bar indicate Student’s *t*-test *p* value. **c** Poly-ubiquitination profile of Ezh1β under scramble and NEDD4 knock-down stable cell line. Scramble/1β-FH and NEDD4 KD/1β-FH indicate stable cell lines: scramble or NEDD4 KD constitutively expressing 1β-FH, respectively. Protein extracts were immunoprecipitated with FLAG and HA agarose beads and purified and ubiquitinated substrates were detected using anti-HA and anti-ubiquitin, respectively. Both scramble/1β-FH and NEDD4 KD/1β-FH stable cell lines were treated without or with H_2_O_2_ were indicated as MT and H_2_O_2_. 10 μM MG-132 was treated for 4 h before protein extraction. Ponceau S staining was used as loading control

We next verified which of the detected E3 ligases would be involved in ubiquitination and degradation of Ezh1β under oxidative stress condition. CHX chasing assay has been widely used to determine degradation rate of target protein [13]. CHX chasing assay confirmed Ezh1β degradation through the 26S proteasome pathway (Additional file 1: Fig. S3a, b). We engineered stable knock-down cell line of each ubiquitin E3 ligase using shRNA hairpin strategy. Analysis of both transcription and protein level of each target clearly show that knock-down efficiency produced by specific shRNA reached at least 60% down regulation (Additional file 1: Fig. S4). Then, we introduced Ezh1β-FH into scramble or E3 ligase knock-down stable cell lines and performed CHX chasing assay to study degradation rate of Ezh1β in absence of each ubiquitin E3 ligase (Additional file 1: Fig. S4b, d, f). We found that knock-down of either HUWE1 or FBXW8 produced minor effects on Ezh1β stability when stable cell lines were challenged by oxidative stress (Additional file 1: Fig. S5). In contrast, when NEDD4 was depleted through shRNA knock-down, degradation of Ezh1β was significantly compromised (Fig. 2a, b). This implies that NEDD4 is the major ubiquitin E3 ligase involved in degradation of Ezh1β under oxidative stress condition. Interestingly, NEDD4 was reported to be involved in muscle atrophy condition [21, 22].

To verify whether NEDD4 indeed plays role as E3 ligase in mediating ubiquitination of Ezh1β and its degradation, we checked ubiquitinated Ezh1β levels in scramble and NEDD4 depletion background under normal and stress conditions (Fig. 2c). Once NEDD4 was removed using shRNA hairpin knock-down in H_2_O_2_-treated cells, increase of Ezh1β ubiquitination pattern was severely compromised (Fig. 2c). We conclude that NEDD4 is the ubiquitin E3 ligase involved in ubiquitination of Ezh1β regulating its degradation when post-mitotic muscle cells are challenged by oxidative stress.

**Fig. 3:**
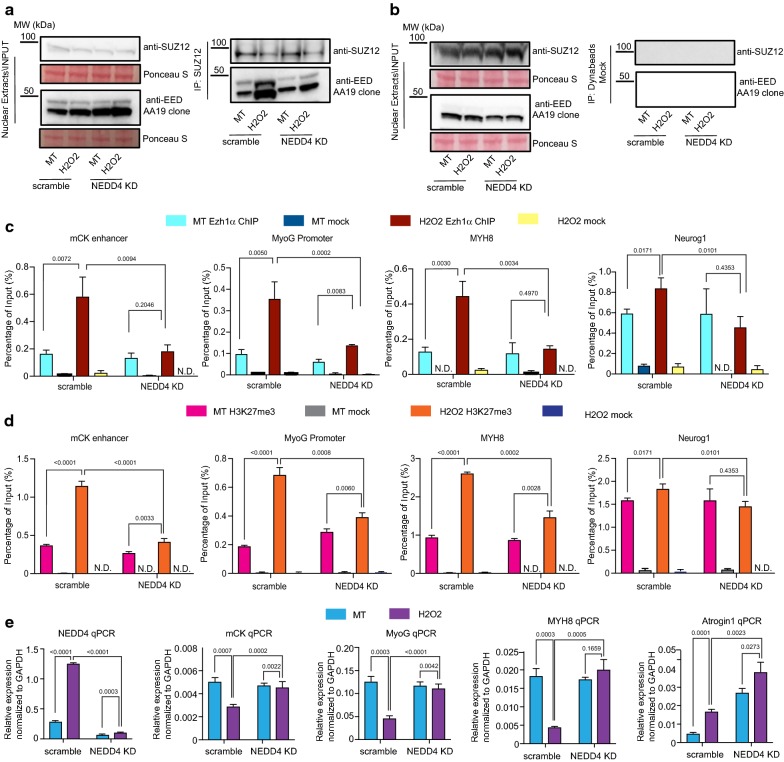
Depletion of NEDD4 compromises assembly of PRC2-Ezh1 complex and H3K27me3 occupancy on mCK, MyoG and MYH8 genomic loci under oxidative stress conditions. **a**, **b** Interaction between SUZ12 and EED was determined in scramble and NEDD4 knockdown stable cell lines under normal and oxidative stress conditions. Nuclear extracts from scramble and NEDD4 KD cell lines under normal and stress conditions were immunoprecipitated with SUZ12 antibody, associated protein complexes were eluted with 2XLDS loading buffer. SUZ12 and EED were detected using anti-SUZ12 and anti-EED. Protein A Dynabeads alone were incubated with nuclear extracts and used as mock control. Ponceau S staining was used as loading control. **c**, **d** ChIP-qPCR analysis of Ezh1α occupancy and H3K27me3 status on genomic loci of mCK enhancer, MyoG promoter, MYH8 and Neurog1. Chromatin immunoprecipitation (ChIP) was performed using chromatin from scramble and NEDD4 KD stable cell lines under normal and oxidative stress conditions against Ezh1α or H3K27me3 antibody. Precipitated DNA were measured using qPCR assay with specific primers corresponding to mCK enhancer, MyoG promoter, MYH8 and NeuroG1 genomic regions. ChIP enrichments are shown as percentage of input. Data were expressed as mean ± SD from three biological replicates. Values above each bar indicate Student’s *t*-test *p* value. **e** Transcription level of NEDD4, mCK, MyoG, MYH8 and Atrogin1 were analyzed using RT-qPCR in scramble and NEDD4 KD stable cell lines under normal and oxidative stress conditions. Data were expressed as mean ± SD from three biological replicates. GAPDH was normalized to get relative expression of each target. Values above each bar indicate Student’s *t*-test *p* value

Next, we attempted to verify how NEDD4-mediated dynamic ubiquitination pattern of Ezh1β occurs under sudden changing physiological conditions. In a previous study it was reported that expression of NEDD4 is upregulated following denervation-induced muscle atrophy condition [22]. Thus, transcription and protein levels of NEDD4 were checked. We found that NEDD4 transcription level increases dramatically (Fig. 3c), followed by a slight upregulation of NEDD4 protein level (Additional file 1: Fig. S6). Ubiquitination of Ezh1β through NEDD4 requires association with each other, therefore we investigated the interaction between Ezh1β and NEDD4 under normal and stress conditions. Co-immunoprecipitation assay showed that both endogenous and exogenous Ezh1β-FH did not exhibit significant association dynamic changes pattern with NEDD4 under normal and atrophy conditions (Additional file 1: Fig. S6). Overall, although NEDD4 transcription level increased under oxidative stressed mimic atrophy condition, its protein level and association with Ezh1β did not exhibit dramatic changes upon oxidative challenging conditions. These data imply that additional mechanisms control NEDD4-dependent Ezh1β ubiquitination under oxidative stress condition.

### Both canonical PRC2-Ezh1 complex assembly and H3K27me3 signature were compromised after depletion of NEDD4

Our previous report showed that degradation of Ezh1β will release EED from cytosol to the nucleus, to facilitate canonical PRC2-Ezh1 repressive complex assembly [12]. Our data have demonstrated that NEDD4 is required for Ezh1β ubiquitination and degradation. Therefore, we determined the interaction between SUZ12 and EED in the nucleus in scramble and NEDD4 knock-down background under normal and stress conditions. Indeed, both EED isoforms exhibited enhanced association pattern with SUZ12 in nucleus in scramble cell lines under oxidative stress condition (Fig. 3a, b). Moreover, dramatic decrease in interaction between SUZ12 and EED was observed when NEDD4 was depleted under oxidative stress condition (Fig. 3a, b).

Next, we verified how defective assembly of canonical PRC2-Ezh1 complex would influence Ezh1α occupancy and H3K27me3 status on muscle specific marker genes loci [12]. We checked Ezh1α occupancy and H3K27me3 status on mCK enhancer, MyoG promoter and MYH8 genomic region using ChIP-qPCR. Compared with the normally increased Ezh1α binding profile and H3K27me3 pattern on those loci in oxidative stress condition, both Ezh1α occupancy, H3K27me3 levels and silenced state of the same genomic region were dramatically affected in H_2_O_2_ treated NEDD4 knock-down cells (Fig. 3c–e).

### Serine 560 phosphorylation of Ezh1β is required for its ubiquitination and degradation

**Fig. 4:**
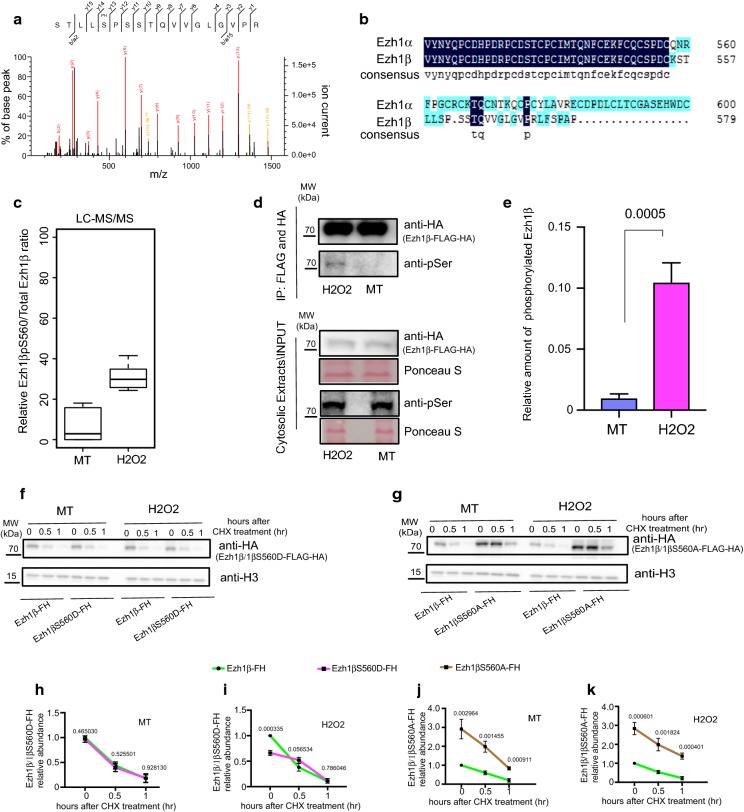
Ezh1β Serine 560 phosphorylation is required for Ezh1β poly-ubiquitination and degradation upon oxidative stress. **a** Mass spectra showing phosphorylation site of Ezh1β localized at Serine 560 amino acid. **b** Sequence alignment between C terminal of Ezh1α and Ezh1β protein sequence. Consensus sequence was highlighted. **c** LC–MS/MS quantification of Ezh1βS560 phosphorylation levels under normal and oxidative stress conditions, *p*-value = 0.001702. **d** Increased Ezh1β Serine 560 phosphorylation status was validated through Immunoprecipitation of Ezh1β-FH coupled with immunoblotting through anti-HA, anti-p-Ser, respectively. Ponceau S staining was used as loading control. **e** Quantification of phosphorylated Ezh1β Serine 560 level presented in **d**, *p*-value = 0.0005. CHX chasing assay of Ezh1β-FH, Ezh1βS560D-FH (**f**) and Ezh1βS560A-FH (**g**) under normal and oxidative stress conditions. Ezh1β-FH, Ezh1βS560D-FH and Ezh1βS560A-FH mean different stable cell lines expressing wild-type Ezh1β, point mutation form Ezh1βS560D and Ezh1βS560A fusion with FLAG and HA. Total proteins were extracted from indicated stable cell lines under normal and oxidative stress conditions. 100 mg/ml CHX was added during last hour before protein extraction and treated as indicated time points. Immunoblotting was used to detect remaining protein levels of Ezh1β or Ezh1β mutant forms with anti-HA. Anti-actin was used as loading control. **h**–**k** Quantification of remaining Ezh1β-FH, Ezh1βS560D-FH (**h**, **i**) and Ezh1βS560A-FH (**j**, **k**) shown in **f** and **g**, respectively. MT means myotube stage sample and H_2_O_2_ means myotube sample stressed with 100 μM H_2_O_2_ for 24 h. Actin protein abundance was normalized and data were expressed as mean ± SD from three biological replicates. Values above each bar indicate Student’s *t*-test *p* value

**Fig. 5:**
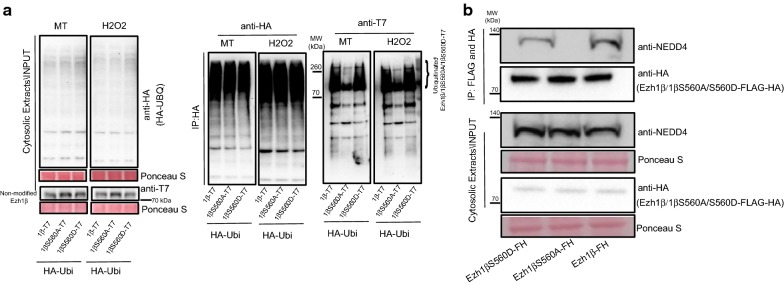
Ezh1β Serine 560 phosphorylation is required for interaction between Ezh1β and NEDD4. **a** Poly-ubiquitination profiles of Ezh1β-T7, Ezh1βS560A-T7 and Ezh1βS560D-T7 under normal and oxidative stress conditions. HA-ubiquitin/Ezh1β-T7, HA-ubiquitin/Ezh1βS560A-T7 and HA-ubiquitin/Ezh1βS560D-T7 indicate C2C12 cell lines co-expressing HA-Ubiquitin and different forms of Ezh1β-T7 fusion proteins. Total proteins were extracted from these indicated stable cell lines. Ubiquitinated total proteins and ubiquitinated different Ezh1β forms were immunoprecipitated with HA agarose beads. Samples were eluted with HA peptide and running SDS-PAGE, anti-HA and anti-Ezh1β were used to detect total ubiquitinated proteins and ubiquitinated Ezh1β or mutant Ezh1β forms. 100 μM MG-132 was added and treated for 4 h before protein extraction. Ponceau S staining was used as loading control. **b** Interaction between NEDD4 and different forms of Ezh1β: Ezh1β, Ezh1βS560A and Ezh1βS560D. Cytosolic proteins were extracted and tandemly immunoprecipitated with Flag and HA agarose beads. NEDD4 antibody was used for immunoblotting analysis. Ponceau S staining was used as loading control

Previous studies reported that phosphorylation of Ezh2 has close and positive role in enhancing ubiquitination and degradation of Ezh2 [15, 18]. Therefore, we asked whether some potential phosphorylation sites might exist in Ezh1β. After careful scanning our MS spectra profile of peptides derived from Ezh1β, serine 560 was identified as one novel phosphorylation site specifically existing in Ezh1β (Fig. 4a, b). Ubiquitination of Ezh1β is enhanced under oxidative stress condition, therefore we sought to check whether serine 560 phosphorylation of Ezh1β would increase upon same condition. Indeed, compared with normal myotube condition, serine 560 phosphorylated form of Ezh1β increased significantly upon stress treatment **(**Fig. 4c–e**)**. Next, to decipher whether Serine 560 is involved in degradation of Ezh1β, we used constitutively activated phosphorylation mutant Ezh1βS560D and Ezh1β resistant mutant Ezh1βS560A. CHX chasing assay, showed that rapid degradation patterns of Ezh1β and Ezh1βS560D were lost when serine was changed to Ala under both normal and oxidative stress conditions **(**Fig. 4f–k**)**. Next, we tried to investigate mechanism mediating Ezh1β phosphorylation-dependent ubiquitination. Actually, many possible mechanisms have been proposed and investigated to understand relationship between phosphorylation and ubiquitination occurring at the same target [23]. Our in vivo ubiquitination assay of wild-type Ezh1β, phosphorylation deficient form of Ezh1βS560A and constitutive active mimic phosphorylation form of Ezh1βS560D, clearly showed that phosphorylation facilitates ubiquitination of Ezh1β (Fig. 5a). Therefore, we checked whether phosphorylation of Ezh1β would enhance interaction between substrate and ubiquitin E3 ligase. Stable cell line constitutively expressing Ezh1β, Ezh1βS560A and Ezh1βS560D were used to pull down different forms of Ezh1β and determine their interactions with NEDD4. We found that both Ezh1β and Ezh1βS560D could be immunoprecipitated with NEDD4 under oxidative stress condition, whereas Ezh1βS560A could not interact with NEDD4 under stress condition (Fig. 5b). We conclude that increased serine 560 phosphorylation of Ezh1β level upon oxidative stress would facilitate interaction between NEDD4 and Ezh1β, which will enhance ubiquitination and degradation of Ezh1β.

### Ubiquitin E3 ligase HUWE1 controls Ezh1α homeostasis under normal conditions

**Fig. 6:**
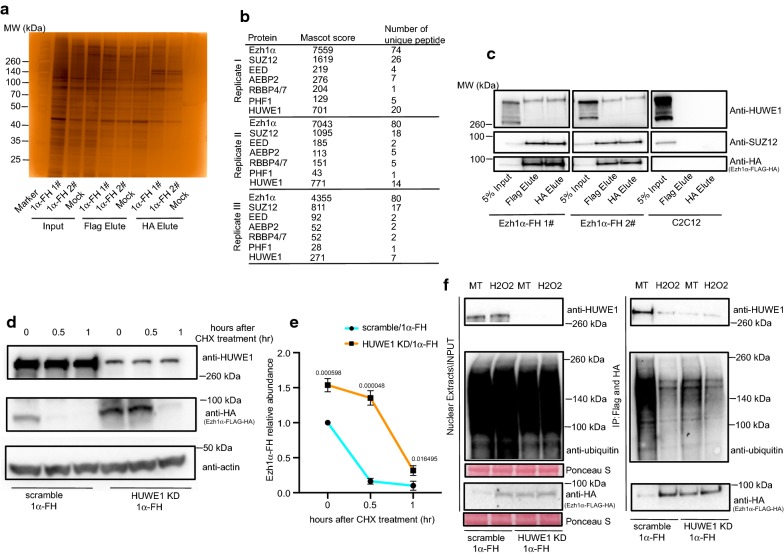
HUWE1-mediated changes of Ezh1α through regulating diverse poly-ubiquitination profiles of Ezh1α under normal and oxidative stress conditions. **a** Ezh1α-FH-associated protein complexes were tandemly affinity purified from nuclear extracts of C2C12 stable cell line which expresses C-terminally FLAG-HA tagged Ezh1α. Flag and HA elutes indicate samples eluted with FLAG and HA peptides, respectively. Flag and HA elute samples were separated by SDS-PAGE and silver stained. Nuclear protein extracts from C2C12 wild-type cell line were used as mock. Ezh1α-FH expressing C2C12 and normal C2C12 myotube samples, after changing differentiation medium for 2 days were used for nuclear protein extraction. **b** Tandem affinity enriched Ezh1α-FLAG-HA associated proteins were identified by MS analysis. For each annotated protein, number of unique peptides and MASCOT score were listed. Data from at least three independent biological replicates data are presented. **c** Interaction among Ezh1α and SUZ12 ubiquitin E3 ligase HUWE1 were validated through Co-immunoprecipitation (Co-IP) assay. Ezh1α-FH was immunoprecipitated from nuclear extracts of two independent C2C12 stable cell line expressing Ezh1α-FH (#1 and #2). Wild-type C2C12 cell line was used as mock control. Ezh1α-FH expressing C2C12 and normal C2C12 myotube samples, after changing differentiation medium for 2 days, were used for protein extraction. Immunoblot analyses were performed with anti-HUWE1, anti-SUZ12, and anti-HA. **d** CHX chasing assay were performed in scramble/Ezh1α-FH and HUWE1 KD/Ezh1α-FH cell lines under normal myotube samples. 100 μg/ml cycloheximide (CHX) was added at indicated time points. Total proteins were extracted and immunoblot analysis was performed using anti-HUWE1 and anti-HA, anti-actin was used as loading control. **e** Quantification of remaining Ezh1α-FH protein percentage in **d**. Relative Ezh1α-FH was quantified in comparison remaining Ezh1α-FH with initial total protein amount at indicated CHX treatment time points. Data were expressed as mean ± SD from three biological replicates. ImageJ software was used to determine protein abundance. Values above each bar indicate Student’s *t*-test *p* value. **f** Dynamic interaction between HUWE1 and Ezh1α under normal and stress conditions. Ezh1α-FH was enriched with FLAG and HA agarose beads, then FLAG and HA peptide were used to elute immunoprecipitated protein partners. Interaction between HUWE1 and Ezh1α, ubiquitination profile of Ezh1α were determined by anti-HUWE1 and anti-ubiquitin. Ponceau S staining was used as loading control

We have characterized the mechanism regulating the stability and degradation of Ezh1β. In this context, another important question is about homeostasis of Ezh1α. To address this question, we firstly established C2C12 cell line expressing Ezh1α-FLAG-HA (Additional file 1: Fig. S7). Taking advantage of this stable cell line, we firstly checked Ezh1α degradation kinetics by using CHX chasing assay. CHX chasing assay clearly showed that almost 80% of Ezh1α was degraded after blocking translation process within half hour by CHX, partially restored when 26S proteasome signaling pathway was blocked by MG-132 (Additional file 1: Fig. S8). Interestingly, in comparison with degradation curve of Ezh1β, rate of Ezh1α degradation exhibited a steeper curve under normal condition. Thus we sought to identify potential interacting protein partners involved in Ezh1α degradation process. Tandem affinity purification strategy was performed to identify associated protein components with Ezh1α under normal condition. Our MS analysis shown that not only PRC2-Ezh1 core components SUZ12 and EED were present, but some other ancillary components such as PHF1, AEBP2 and RBBP4/7 could also be detected using this strategy (Additional File 4: Datasheet 3; Fig. 6a, b).

Intriguingly, HUWE1 ubiquitin E3 ligase was captured in our Ezh1α TAP assay. To validate interaction between HUWE1 and Ezh1α, co-immunoprecipitation assay was performed. We found that both SUZ12, used as positive control for this assay, and HUWE1 are interacting partners with Ezh1α in post-mitotic muscle myotube (Fig. 6c).

We demonstrated that ubiquitin E3 ligase HUWE1 could interact with Ezh1α (Fig. 6b, c), thus, we wanted to know whether HUWE1 would modulate stability of Ezh1α. To this we firstly established scramble and HUWE1 knock-down stable cell lines, expressing Ezh1α-FH in these two different genetic backgrounds (Additional file 1: Fig. S4**)**. Then, we checked Ezh1α degradation pattern changes in presence or absence of HUWE1. CHX chasing assay performed in scramble and HUWE1 knock-down background demonstrated that Ezh1α degradation pattern in scramble background was strikingly restored in the absence of HUWE1 condition (Fig. 6d, e). Based on this observation, we reasoned that ubiquitination of Ezh1α could be affected due to depletion of HUWE1. To address this question, we enriched for Ezh1α using tandem affinity precipitation and checked ubiquitination profiles of Ezh1α. When scramble stable cell lines were challenged with oxidative stress and expression of HUWE1 was reduced using shRNA knock-down strategy, decreased ubiquitination profile of Ezh1α was strikingly lost (Fig. 6f).

We conclude that ubiquitin E3 ligase HUWE1 is required for degradation of Ezh1α. In contrast, under stress conditions Ezh1α would be stabilized through reduced ubiquitination (Fig. 6f**)**. These observations led us to ask whether either dynamic changes of HUWE1 protein level or interaction between HUWE1 and Ezh1α stoichiometry would contribute to dynamic ubiquitination of Ezh1α. No significant changes were observed in HUWE1 levels between normal and stress conditions in scramble cell lines **(**Fig. 6f**)**. However, when we immunoprecipitated Ezh1α and checked amount of HUWE1 associated with Ezh1α the amount of HUWE1 associated with Ezh1α decreased dramatically in oxidative stress. Meanwhile, we also found that ubiquitinated Ezh1α level decreased as well and this contributed to stabilize Ezh1α. Moreover, in HUWE1 knock-down cell line, dynamic ubiquitinated profile changes pattern was lost in keeping with the silencing role of Ezh1α and H3K27m3 in response to oxidative stress **(**Fig. 6f**)**.

## Discussion

Our findings unveil a novel signal-dependent mechanism where fine regulation of compartment specific levels of Ezh1β components is essential to allow PRC2 mediated adaptive function in skeletal muscle cells.

Our data identify the two distinct and specific E3-ubiquitin ligases that control abundance of Ezh1β in the cytoplasm and the stability of Ezh1α in the nucleus as key factors to control appropriate PRC2-Ezh1 activity in terminally differentiated cells. That is, low abundance of Ezh1α pool would provide adequate Ezh1α level to maintain canonical (silencing) and non-canonical (Pol II related) activity under normal condition. However, upon oxidative stress, PRC2-Ezh1 silencing function becomes necessary and prevalent, thus PRC2-Ezh1 complex needs to be efficiently assembled and by this to exert canonical H3K27m3 mediated, adaptive function. To this, EED would be released from Ezh1β-EED cytosol complex, while down regulation of Ezh1α would be prevented to guarantee that functional PRC2 complex formation.

The other important aspect is signaling. Phosphorylation-dependent ubiquitination of Ezh2 was previously reported, and many phosphorylation sites were identified [19, 24]. Protein sequence between Ezh1β and Ezh1α is exactly the same except a short C terminal motif. However, in our MS spectra we did not identify any phosphorylation similar site to Ezh2. Surprisingly, only one specific phosphorylation site was identified from Ezh1β C terminal specific sequence. Therefore this phosphorylation-dependent ubiquitination confers signal specificity to Ezh1β environmental sensor function.

Oxidative stress condition and ROS production are very important indicator or inducer for many different types of tumor [25, 26]. In this context, the elucidation of the presented mechanism controlling the activity of PRC2-Ezh1α/β sheds light on and underlines the importance of plasticity in epigenetic control of cell homeostasis.

## Conclusions

In this study we report about the identification of ubiquitin E3 ligases NEDD4 and HUWE1 mediating dynamics of PRC2-Ezh1α/β pathway, shedding light on novel mechanistic aspects of PcG biology and adaptive stress response. Moreover, we identify Serine 560 phosphorylation of Ezh1β as a key target required for its signal-dependent ubiquitination and degradation upon oxidative stress in skeletal muscle.

## Materials and methods

### Cell culture and treatments

C2C12 mouse skeletal myoblasts (ATCC; CRL-1772) were grown in Dulbecco’s modified Eagle’s medium (DMEM) (4.5 g/l d-glucose and Glutamax) (GIBCO) and 10% fetal bovine serum (FBS; GIBCO) with penicillin–streptomycin supplement, according to standard protocols. HEK293T (ATCC; CRL-3216) and Phoenix-Eco (ATCC; CRL-3214) were cultured in similar condition like mouse C2C12 plus 1 mM sodium pyruvate. When C2C12 reached 90–95% confluence, it was differentiated to myofibers in DMEM and 2% horse serum (GIBCO) with penicillin–streptomycin supplement.

Where indicated, cells were treated with MG-132 (Sigma, 10 μM), cycloheximide (CHX, Sigma, 100 μg ml^−1^). For in vivo ubiquitination assay, MG-132 was added and treated for 4 h before protein extraction. For CHX chasing assay, CHX was added and treated as indicated time under normal myotube or stressed myotube. Oxidative stress was induced following previously described protocol [12].

### Plasmids

For Ezh1α-FLAG-HA, Ezh1β-FLAG-HA, EED_500_-FLAG-HA and EED_441_-FLAG-HA, full-length CDS without stop codon were amplified with corresponding primers (Additional file 5: Table S1) and ligated into pJET1.2 (Thermo Fisher Scientific) vector for Sanger sequencing. Sanger sequencing confirmed inserts were cut with XhoI/NotI and finally ligated into pOZ-C-FH vector.

For Ezh1α-2XT7, Ezh1β-2XT7, Ezh1βS560A-2XT7 and Ezh1βS560D-2XT7, full-length CDS containing stop codon were amplified using indicated primers listed in Appendix Table EV1, then, similar strategy was used to clone inserts into pOZ-C-FH vectors.

For Lenti-HA-Ubi was purchased from Addgene (Plasmid, #74218). For pOZ-HA-Ubi, HA-Ubi was amplified from Lenti-HA-Ubi and cloned into pJET1.2 (Thermo Fisher Scientific) for Sanger sequencing, then finally cloned into pOZ-C-FH vector.

### Plasmid transfection, retrovirus or lentivirus packaging and infection

To package retrovirus, Phoenix-Eco (ATCC;CRL-3214) was transfected using Lipofectamine 2000 (Thermo Fisher Scientific) according to standard protocol. Transfection medium was changed to virus collection medium (DMEM plus 5% FBS) after 8 h of lipofectamine transfection. After 48 h, virus collection medium containing retrovirus was filtered with 0.45 μm filter and be ready for titer assay or transduction. Lentivirus production was performed in HEK293T (ATCC; CRL-3216) using 3rd Generation Packaging Mix kit (Abmgood) following commercially provided protocol. Validated retroviral and lentiviral vectors containing GFP protein were used as positive control during lipofectamine-mediated transfection process.

Freshly prepared and tittered retrovirus or lentivirus was used to infect C2C12 mouse skeletal myoblasts (ATCC; CRL-1772), 8 μg ml^−1^ polybrene was added during infection procedure. After 8 h, fresh growth medium was added to replace infection medium, after that, C2C12 was allowed to grow another 24–48 h before they reach 80% confluence. Then, positive cells were selected using Anti-CD25 beads (Invitrogen) for retrovirus infected cells, or selected using 1.6 μg ml^−1^ for lentivirus transduced positive cells.

pLKO shRNA lentivirus to target HUWE1, NEDD4 and FBXW8 were purchased from Sigma: HUWE1 #1 (TRCN0000092554), HUWE1 #2 (TRCN0000092555), NEDD4 #1 (TRCN0000092436), NEDD4 #2(TRCN0000092437), FBXW8 #1 (TRCN0000012731), FBXW8 #2(TRCN0000012732).

### Protein extraction for co-immunoprecipitation and tandem affinity purification

Cytosolic and nuclear extracts were prepared Extracts were prepared using our previous protocol with minor modifications^23^. Briefly, cells were lysed in cytosolic extraction buffer (50 mM Tris–HCl, pH 8, 150 mM NaCl, 0.5 mM EDTA, 0.5% Triton X-100, 5% glycerol). The nuclei were collected at 1500 g and 4 °C, and the supernatant was stored as cytosolic extracts. Nuclei were washed three times in cytosolic extraction buffer and suspended in nuclear extraction buffer (50 mM Tris–HCl, pH 8, 50 mM NaCl, 0.5 mM EDTA, 0.5% Triton X-100, 5% glycerol), sonicated (BRANSON A250 with a 3.2-mm tapered microtip; two cycles of 30 s at 20% amplitude, 50% of duty cycle). Debris was pelleted at 16,380 g and 4 °C, and the supernatant was used for nuclear fraction extracts. Before IP, NaCl concentration would be adjusted to 150 mM.

For Co-IP, each IP was set up with 2 mg of protein in a final volume of 700 μl at a final concentration of 150 mM NaCl; then 7 μg of the appropriate primary antibodies were added and incubated with protein extracts overnight at 4 °C on the wheel. The immunocomplexes were then recovered with 70 μl (1/10 of IP volume) of magnetic Dynabeads (Protein A for primary antibody produced in Rabbit/Protein G for primary antibody produced in mice; Invitrogen) and washed with wash buffer (50 mM Tris–HCl, pH 8, 200 mM NaCl, 0.5 mM EDTA, 0.5% Triton X-100, 5% glycerol) four times, each time wash was carried out for 5 min with rotation at 4 °C. Immuno-precipitates were eluted with 2XLDS loading buffer at 95 °C for 5 min. The eluted immuno-precipitates were loaded on Bolt Bis–Tris precast gel (Invitrogen) and subjected to western blotting analysis. A list of antibodies used is provided in Appendix Table EV2.

For TAP, tagged proteins were immunoprecipitated with anti-Flag M2-agarose (Sigma), and eluted with Flag peptide (0.2 mg/ml). Further affinity purification was performed with anti-HA antibody-conjugated agarose (Pierce), and eluted with HA peptide (0.2 mg/ml). The HA and Flag peptides were prepared as 5 mg/ml stock in 50 mM Tris–Cl (pH 8.5) and 150 mM buffer, then diluted to corresponding concentration in TGEN 150 buffer (20 mM Tris at pH 7.65, 150 mM NaCl, 3 mM MgCl_2_, 0.1 mM EDTA, 10% glycerol, 0.01% NP40). Between each step, beads were washed in TGEN 150 buffer three times. Complexes were resolved by SDS-PAGE and stained using the SilverQuest Silver staining kit (Invitrogen).

### RNA preparation and qPCR

Total RNA was extracted with TRI Reagent (Sigma) according to manufacturer’s instructions. cDNA was prepared starting at 1 μg of RNA from each sample with a QuantiTect reverse-transcription kit (Qiagen). Real-time PCR analyses were carried out using SsoAdvanced™ Universal SYBR^®^ Green Supermix (BioRad) and analyzed in CFX96 Touch™ Real-Time PCR Detection System (BioRad). The primer sequences are provided (Additional file 5: Table S1).

### Chromatin immunoprecipitation (ChIP) and qPCR

Cells were cross-linked in 1% formaldehyde (Thermo Fisher Scientific, 28906) for 10 min at room temperature. Cross-linked cells were lysed in lysis buffer 1 (50 mM HEPES KOH, pH 7.5, 10 mM NaCl, 1 mM EDTA, 10% glycerol, 0.5% NP-40, 0.25% Triton X-100) overnight. Nuclei were collected, washed in lysis buffer 2 (10 mM Tris–HCl, pH 8, 200 mM NaCl, 1 mM EDTA, 0.5 mM EGTA) and lysed in lysis buffer 3 (10 mM Tris–HCl, pH 8, 100 mM NaCl, 1 mM EDTA, 0.5 mM EGTA, 0.1% Na-deoxycholate, 0.5% N-lauroylsarcosine). Freshly prepared 1 × protease inhibitor cocktail was added into all lysis buffers. Chromatin was sheared (BRANSON A250 with a 3.2-mm tapered microtip; four to five cycles of 2 min at 20% amplitude, 50% of duty cycle). In each IP reaction, 100 μg of chromatin DNA equivalents (DNA concentration detected at Nanodrop) were incubated overnight with 5–8 μg of antibodies. The immunocomplexes were recovered with magnetic Dynabeads (Protein A; Invitrogen) for 2 h and washed on the wheel at 4 °C for 5 min with Low-Salt (LS) wash buffer (0.1% SDS, 2 mM EDTA, 1% Triton X-100, 20 mM Tris–HCl, pH 8, 150 mM NaCl) and High-Salt (HS) wash buffer (0.1% SDS, 2 mM EDTA, 1% Triton X-100, 20 mM Tris–HCl, pH 8, 500 mM NaCl). Then, LS and HS buffers wash were repeated one more time. Final wash was carried out with TE buffer plus 150 mM NaCl twice. Precipitated DNA was eluted using elution buffer (50 mM Tris–HCl, pH 8, 10 mM EDTA, 1% SDS) at 65 °C for 15 min. For de-cross-linking, all eluted samples were incubated at 65 °C overnight. Chromatin was digested with RNase A (0.2 mg/ml) and proteinase K (0.2 mg/ml), and DNA was purified for qPCR analysis. H3K27me3 ChIP results are expressed as percentage of input. A list of oligos and antibodies used are provided in Additional file 5: Table S1, Additional file 6: Table S2.

### Immunofluorescence

Stable C2C12 cell lines constitutively expressing Ezh1α-FH or Ezh1β-FH were cultured for myoblast or differentiated to myotubes were fixed with 4% PFA for 15 min at room temperature, permeabilized with 0.1% Triton X-100 in PBS for 10 min, and blocked with 1% BSA solution. Primary antibody staining was performed for 1 h at room temperature in a 1% BSA solution at dilutions of 1:200 for HA (Roche; 3F10) and 1:500 for MHC/MF-20 (DSHB; 051320). After three times washes with 0.1% PBS, secondary antibody staining was carried out at room temperature in a 1% BSA solution (1:500). Secondary antibodies conjugated Alexa Fluor 488 (Invitrogen, A-11006) or Alexa Fluor 568 (Invitrogen, A-11031). Mounting medium containing DAPI (Sigma, F6057) was used to counterstain nuclei localization. Images were obtained with a Leica TCS SP5 confocal microscope with an HCX PL APO 63.0×/1.40-NA oil-immersion objective.

### Protein digestion and peptide fractionation

HA peptide eluted samples from TAP assay were diluted in 8 M urea in 0.1 M Tris–HCl followed by protein digestion with trypsin according to the FASP protocol [27]. After an overnight digestion peptides were eluted from the filters with 25 mM ammonium bicarbonate buffer. Eluted peptide was processed desalting step by using Sep-Pag C18 Column (Waters) based on manufacture’s instruction.

### Liquid chromatography–mass spectrometry (LC–MS) analysis and MS data analysis

The peptide mixture was measured on a Q Exactive HF mass spectrometer (Thermo Fisher Scientific) coupled with an UltiMate™ 3000 UHPLC (Thermo Fisher Scientific). Peptides were separated using an Acclaim PepMap100 C18 column (75 um I.D. X 25 cm, 3 μm particle sizes, 100 Å pore sizes) with a flow rate of 300 nl/min. A 75-minute gradient was established using mobile phase A (0.1% FA) and mobile phase B (0.1% FA in 80% ACN): 5–40% B for 55 min, 5-min ramping to 90% B, 90% B for 5 min, and 2% B for 10-minute column conditioning. The sample was introduced into mass spectrometer through a Nanospray Flex (Thermo Fisher Scientific) with an electrospray potential of 1.5 kV. The ion transfer tube temperature was set at 160 °C. The Q Exactive was set to perform data acquisition in DDA mode. A full MS scan (350–1400 m/z range) was acquired in the Orbitrap at a resolution of 60,000 (at 200 *m/z*) in a profile mode, a maximum ion accumulation time of 100 ms and a target value of 3 × e^6^. Charge state screening for precursor ion was activated. The ten most intense ions above a 2e4 threshold and carrying multiple charges were selected for fragmentation using higher energy collision dissociation (HCD). The resolution was set as 15,000. Dynamic exclusion for HCD fragmentation was 20 s. Other setting for fragment ions included a maximum ion accumulation time of 100 ms, a target value of 1 × e^5^, a normalized collision energy at 28%, and isolation width of 1.8.

The MS RAW files from Q-Exactive HF were converted to.mgf files using Proteome discoverer (V1.4) and analyzed using Mascot (Version 2.4) against mouse database (Uniprot Mus musculus database). The Mascot search results were further processed using Scaffold (Version 4.1, Proteomesoftware Inc., Portland, OR, USA) for validation of protein identification and quantitative assessment. For protein identification, it requires a minimal 99% possibility for protein and with at least one peptide having a possibility greater than 95% according to the PeptideProphet [28] and ProteinProphet [29]. The label-free quantification of proteins and phosphorylation peptides were performed using Maxquant LFQ [30]. Detailed ratio calculation of phosphorylated Ezh1b Serine 560 formula has been described previously [31].

### Quantification of western blots

Band intensity of immunoblots was quantified using ImageJ software. Quantification was calculated by normalization to appropriate indicated internal references. For CHX half-life experiments, the maximum was scaled to 1 by dividing all normalized time points by the normalized control.

### Statistical analysis

Samples were compared using two-tailed, unpaired Student’s *t* test, unless otherwise stated. Error bars were represented by SD± as indicated.

## Supplementary information

**Additional file 1: Fig. S1.** Construction of stable C2C12 cell line constitutively expressing Ezh1β. **Fig. S2.** Increased poly-ubiquitination status of Ezh1β under oxidative stress condition. **Fig. S3.** Degradation of Ezh1β-FH is dependent on 26S proteasome system. **Fig. S4.** HUWE1, NEDD4 and FBXW8 knock-down stable cell line construction. **Fig. S5.** Minor effect of HUWE1 and FBXW8 in regulating stability of Ezh1β under oxidative stress condition. **Fig. S6.** Dynamic interaction between Ezh1β and NEDD4 under normal and oxidative stress conditions. **Fig. S7.** Construction of Ezh1α-FH stable cell line. **Fig. S8.** CHX chasing assay of Ezh1α-FH under normal condition.

**Additional file 2: Datasheet 1.** Mass spectrometry analysis data of low and high molecular weight band for Ezh1 short isoform.

**Additional file 3: Datasheet 2.** Protein interactome list of Ezh1 short isoform through mass spectrometry analysis under oxidative stress condition.

**Additional file 4: Datasheet 3.** Protein interactome list of Ezh1 long isoform through mass spectrometry analysis under normal condition.

**Additional file 5: Table S1.** Oligos sequence information used in this study.

**Additional file 6: Table S2.** Primary antibodies information used in this study.

## Data Availability

The datasets and original source data used and/or analyzed during the current study are available from the corresponding author on reasonable request.

## Abbreviations

TAP: tandem affinity purification
LC–MS: liquid chromatography–mass spectrometry
ChIP: chromatin immunoprecipitation
