# Supplementary material for: Ubiquitin ligases HUWE1 and NEDD4 cooperatively control signal-dependent PRC2-Ezh1α/β-mediated adaptive stress response pathway in skeletal muscle cells
Source: Epigenetics Chromatin. 2019 Dec 19;12:78. doi: 10.1186/s13072-019-0322-5 (PMC6921592; doi:10.1186/s13072-019-0322-5)
Supplement: Supplementary file 1 — Additional file 1: Fig. S1. Construction of stable C2C12 cell line constitutively expressing Ezh1β. Fig. S2. Increased poly-ubiquitination status of Ezh1β under oxidative stress condition. Fig. S3. Degradation of Ezh1β-FH is dependent on 26S proteasome system. Fig. S4. HUWE1, NEDD4 and FBXW8 knock-down stable cell line construction. Fig. S5. Minor effect of HUWE1 and FBXW8 in regulating stability of Ezh1β under oxidative stress condition. Fig. S6. Dynamic interaction between Ezh1β and NEDD4 under normal and oxidative stress conditions. Fig. S7. Construction of Ezh1α-FH stable cell line. Fig. S8. CHX chasing assay of Ezh1α-FH under normal condition. [file 13072_2019_322_MOESM1_ESM.pdf]

Additional File 1 Contents

Fig. S1 Construction of stable C2C12 cell line constitutively expressing Ezh1β.

Fig. S2 Increased poly-ubiquitination status of Ezh1β under oxidative stress condition.

Fig. S3 Degradation of Ezh1β-FH is dependent on 26S proteasome system.

Fig. S4 HUWE1, NEDD4 and FBXW8 knock-down stable cell line construction.

Fig. S5 Minor effect of HUWE1 and FBXW8 in regulating stability of Ezh1β under oxidative stress condition.

Fig. S6 Dynamic interaction between Ezh1β and NEDD4 under normal and oxidative stress conditions.

Fig. S7 Construction of Ezh1α-FH stable cell line.

Fig. S8 CHX chasing assay of Ezh1α-FH under normal condition.

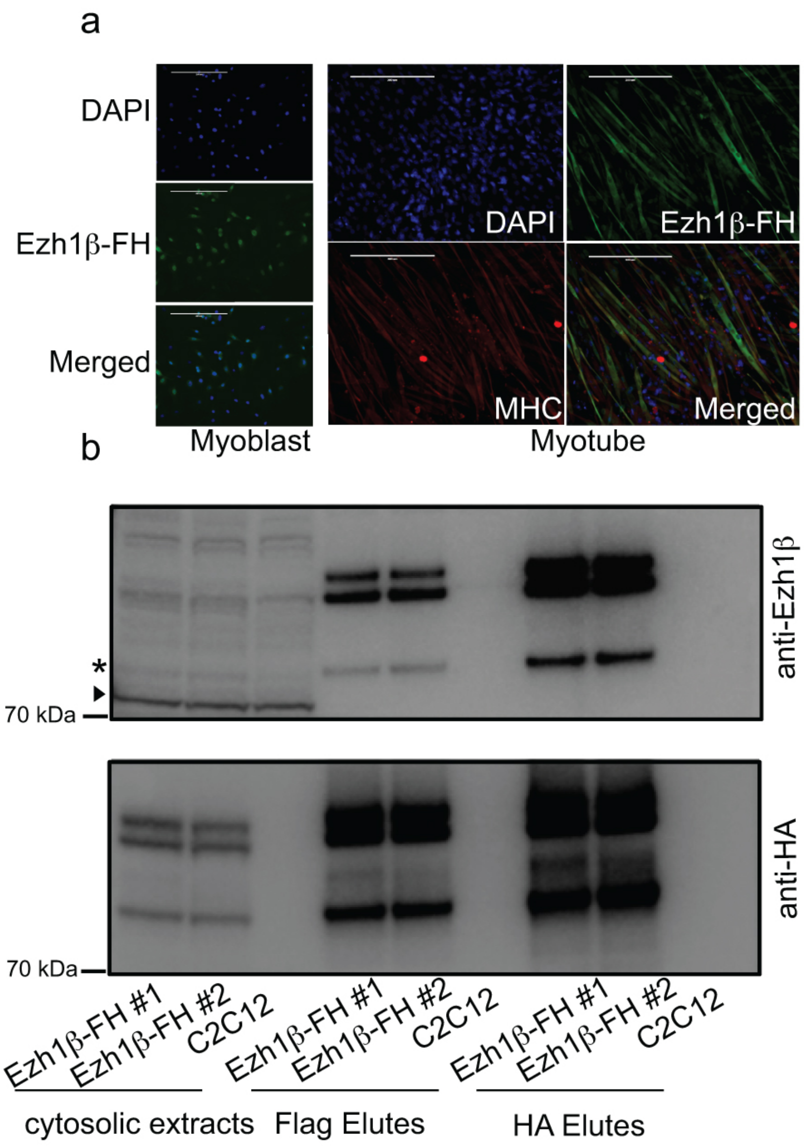

Fig. S1 Construction of stable C2C12 cell line constitutively expressing Ezh1β.

a Stable expression of Ezh1β in C2C12 cell line myoblast (left panel) and myofiber (right panel). In myoblast stage, Ezh1β-FH signal was indicated as green and nuclear location was stained with DAPI; In myotube stage, Ezh1β-FH localization was indicated as green and nuclear location was stained with DAPI, MHC was presented with Red. Ezh1β-FH means fusion protein Ezh1β tagged with tandem FLAG and HA. MHC indicates myosin heavy chain. Scale bar, 200 μm

b Expression level of endogenous Ezh1β and tagged Ezh1β-FH fusion protein.

Immunoblot analysis of endogenous Ezh1β and tagged Ezh1β-FH fusion protein in cytosolic extracts from two independent C2C12 stable cell line expressing Ezh1β-FH (Ezh1β-FH #1 and #2) and C2C12. Anti-Ezh1β and Anti-HA was used in immunoblot analysis. Cytosolic extracts indicate input samples, Flag and HA elutes indicate output samples after elution with Flag peptide and HA peptide respectively. Black star indicates molecular band of exogenous Ezh1β-FH and black triangle indicates location of endogenous Ezh1β.

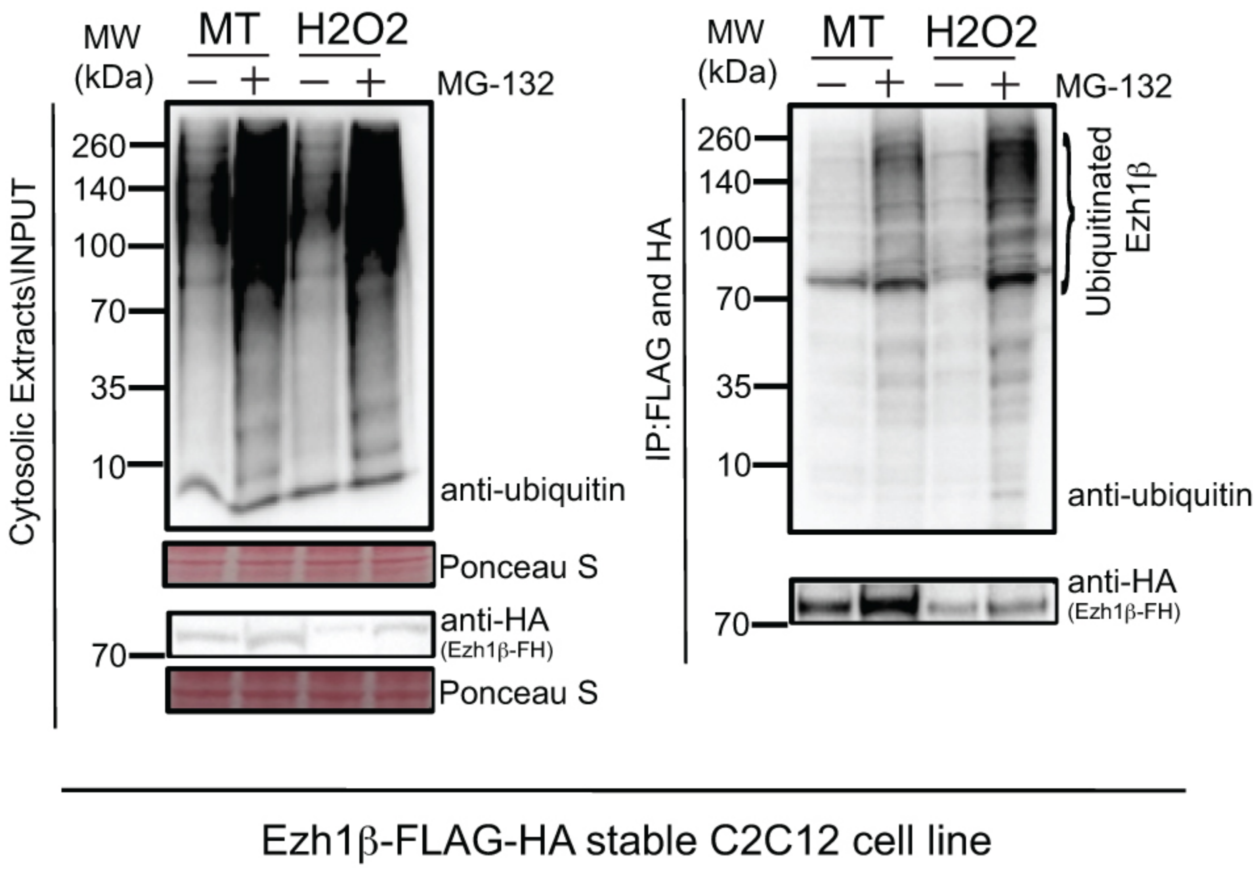

Fig. S2: Increased poly-ubiquitination status of Ezh1β under oxidative stress condition.

Ubiquitinated status of Ezh1β-FLAG-HA in C2C12 cell line under normal and stress conditions. Ezh1β-FLAG-HA were purified with tandem FLAG and HA agarose beads immunoprecipitation coupled with FLAG and HA peptide elution, then, eluted samples were detected with anti-HA and anti-ubiquitin antibody respectively. 10 μM MG-132 was incubated for 4 hours before cytosolic proteins were extracted. Ponceau S indicates loading control. MT means myotube day 1 and H2O2 means myotube 1 treated with 100 μM H2O2 for 24 hours. All experiments have been repeated at least three times.

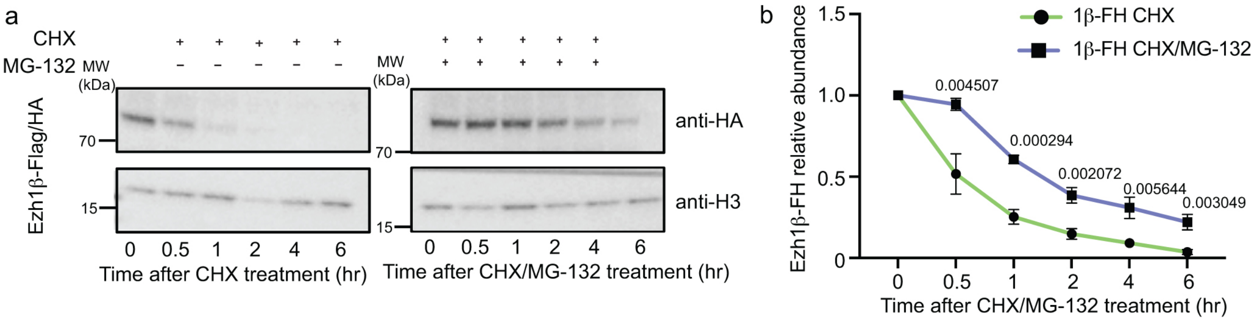

Fig. S3 Degradation of Ezh1β-FH is dependent on 26S proteasome system.

a Total proteins were extracted from stable Ezh1β-FH C2C12 cell line at indicated different time points after treatment with 100 μg/ml cycloheximide (CHX) alone or together with 10 μM MG-132. Immunoblot analysis was performed using anti-HA and anti-H3.

b Percentage of remaining Ezh1β-FH level compared with initial protein level was quantified using ImageJ software.

Data was expressed in (**b**) as means ± SD from three biological replicates. Values above each bar indicate Student's t-test p value.

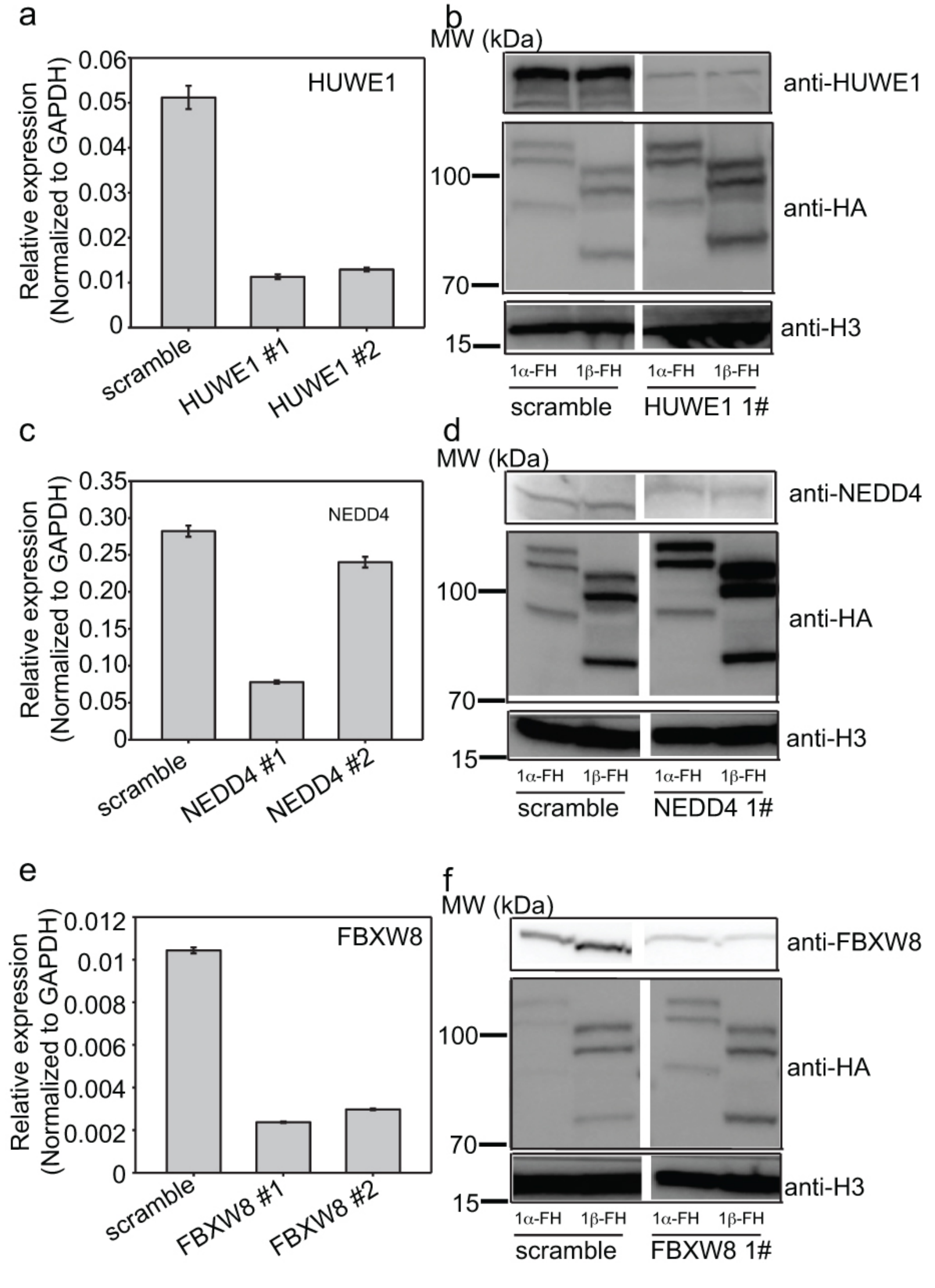

Fig. S4 HUWE1, NEDD4 and FBXW8 knock-down stable cell line construction.

Transcription level of HUWE1 (**a**), NEDD4 (**c**) and FBXW8 (**e**) were detected using RT-qPCR assay in following stable cell line: scramble, HUWE1 knock-down stable cell line

(HUWE1 #1 and #2), NEDD4 knock-down stable cell line (NEDD4 #1 and #2), FBXW8 knock-down stable cell line (FBXW8 #1 and #2). Transcription levels were normalized to GAPDH, Data were expressed as means ± SD from three biological replicates.

Protein abundance of HUWE1(**b**), NEDD4 (**d**) and FBXW8 (**f**) was detected through immunoblot analysis using anti-HUWE1, anti-NEDD4 and anti-FBXW8. Histone 3 was used as loading control.

Ezh1α-FH and Ezh1β-FH was introduced into scramble, HUWE1 (#1), NEDD4 (#1) and FBXW8 (#1) knock-down cell line to generate stable cell line: scramble/Ezh1α-FH, scramble/Ezh1β-FH, HUWE1 #1/ Ezh1α-FH, HUWE1 #1/ Ezh1β-FH, NEDD4 #1/ Ezh1α-FH, NEDD4 #1/ Ezh1αβ-FH and FBXW8 #1/ Ezh1α-FH, FBXW8 #1/ Ezh1β-FH respectively. Ezh1α-FH and Ezh1β-FH protein level were detected using anti-HA Immunoblot analysis.

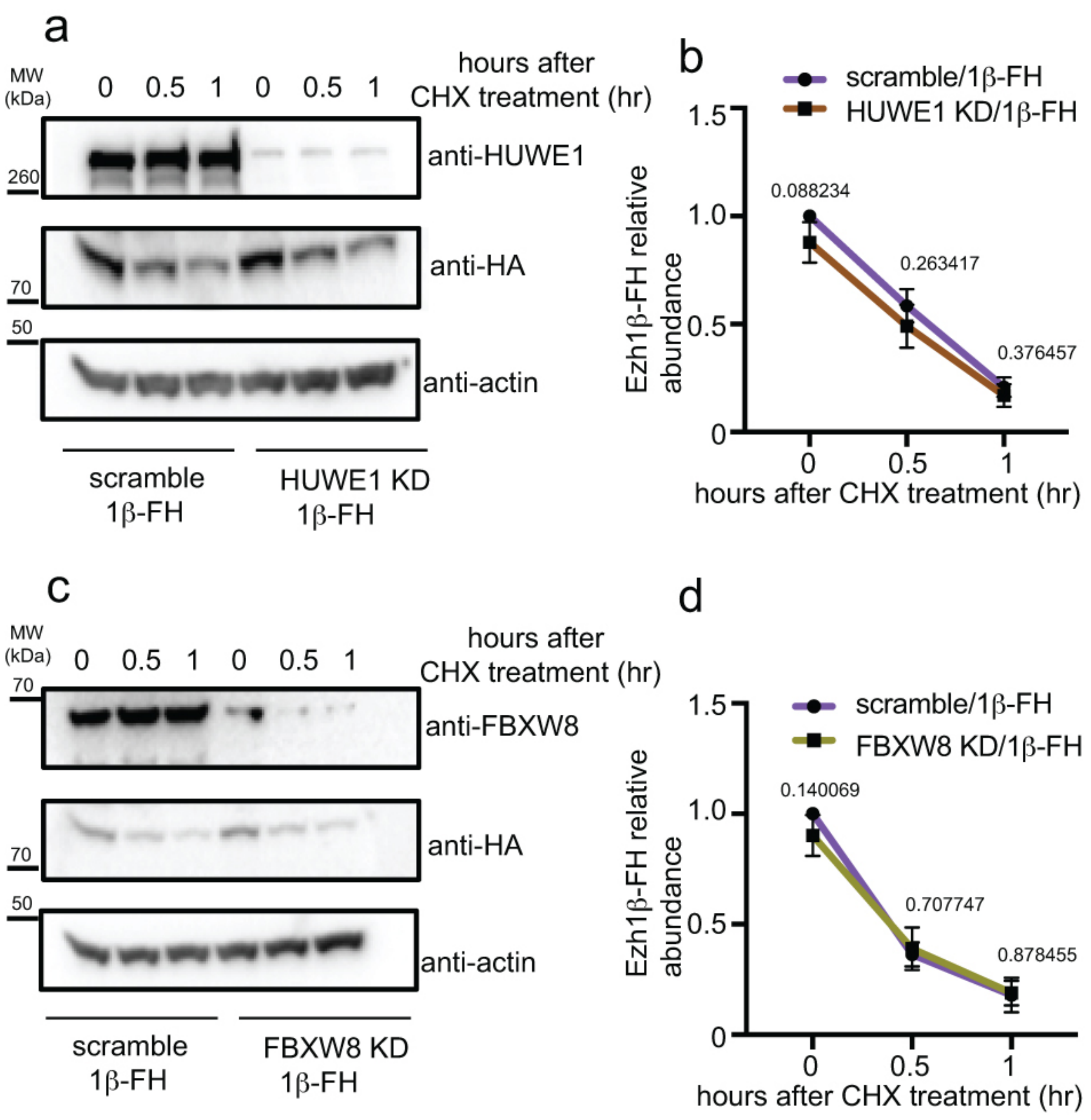

Fig. S5 Minor effect of HUWE1 and FBXW8 in regulating stability of Ezh1β under oxidative stress condition.

Ezh1β-FH degradation rate was determined in scramble and HUWE1 knock-down background (**a**) or FBXW8 knockdown (**c**) through CHX chasing assay. All stable cell lines were treated with 100 μM H2O2 for 24 hours. During last hour of H2O2 treatment, 100

μg/ml cycloheximide (CHX) was added at indicated time points. Total proteins were extracted for immunoblot analysis to check protein level of Ezh1β-FH and HUWE1 (**a**) or Ezh1β-FH and FBXW8 (**c**). Anti-actin was used as loading control. (**b**) and (**d**) were quantification analysis of percentage remaining Ezh1β-FH compared with initial total Ezh1β-FH protein in (**a**) and (**c**) respectively. Data were expressed as means ± SD from three biological replicates. Values above each bar indicate Student's t-test p value.

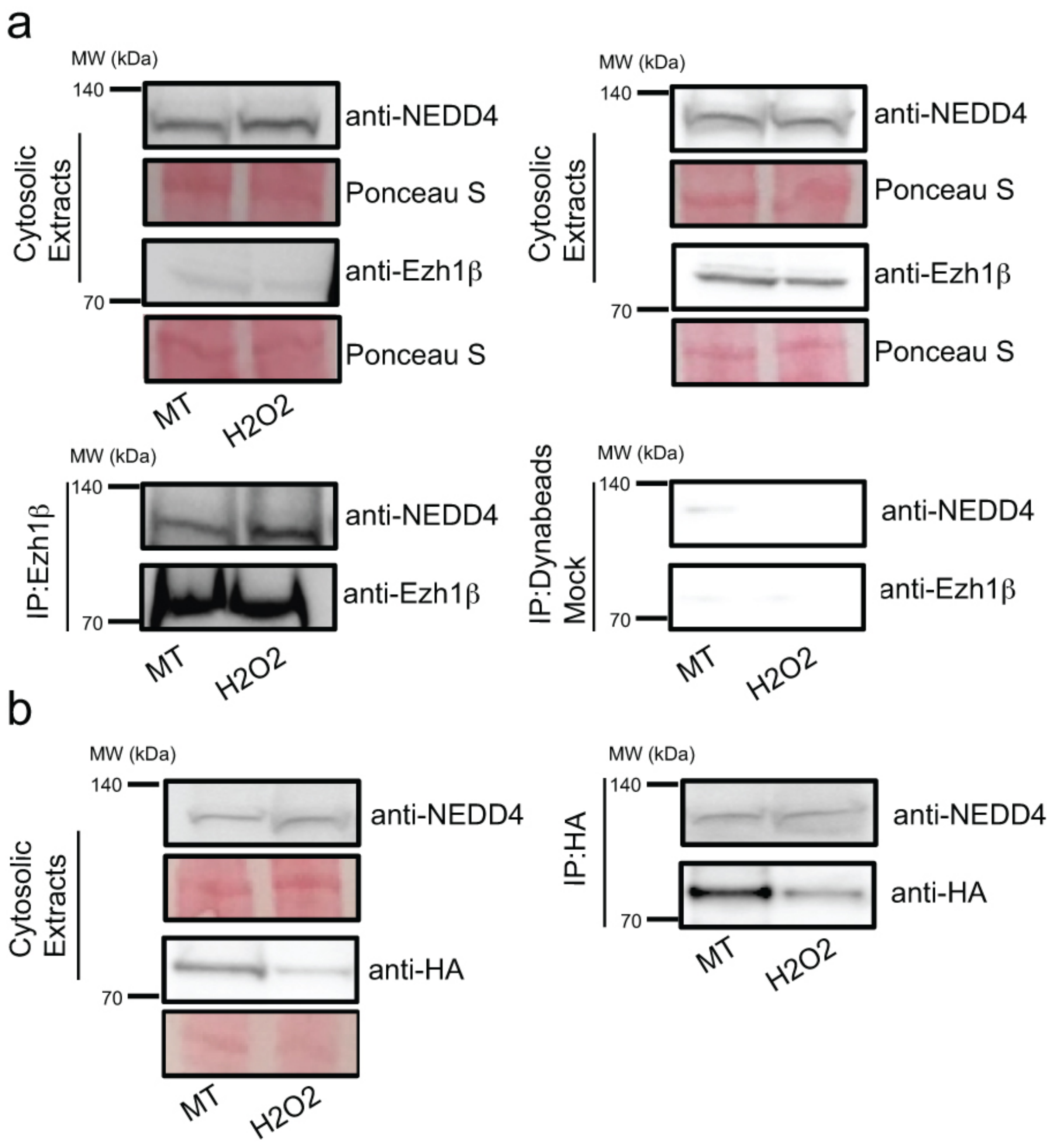

Fig. S6 Dynamic interaction between Ezh1β and NEDD4 under normal and oxidative stress conditions.

a Interaction between endogenous Ezh1β and NEDD4 under C2C12 cells. MT means myotube stage sample and H2O2 means myotube sample stressed with 100 μM H2O2 for 24 hours. Proteins were extracted from C2C12 under indicated different conditions and were immunoprecipitated with Ezh1β antibody. Samples were eluted with 2XLDS loading buffer and immunoblot analysis were performed using anti-Ezh1β and anti-NEDD4. Dynabeads were incubated with protein extracts as mock control. Ponceau S staining was used as loading control.

b Interaction between Ezh1β-FH and NEDD4 in Ezh1β-FH expressing C2C12 stable cell lines under normal and oxidative stress conditions. Proteins were extracted and tandem affinity purified with FLAG and HA agarose beads, HA Elute samples were used for immunoblot analysis with anti-HA and anti-NEDD4. Ponceau S staining was used as loading control.

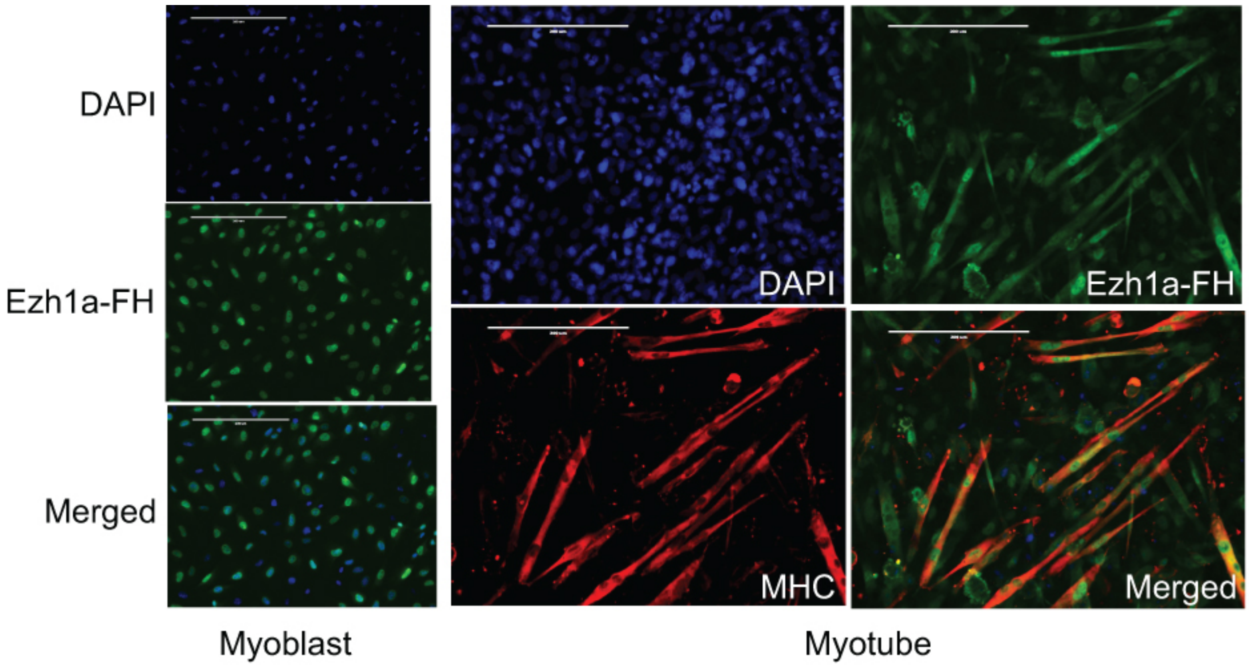

Fig. S7 Construction of Ezh1α-FH stable cell line.

Stable expression of Ezh1α in C2C12 cell line myoblast (left panel) and myofiber (right panel). In myoblast stage, Ezh1α-FH signal is indicated as green and nuclear location was stained with DAPI; In myotube stage, Ezh1α-FH localization is indicated as green and nuclear localization was stained with DAPI, MHC is presented with Red. Ezh1α-FH means fusion protein Ezh1α tagged with tandem FLAG and HA. MHC indicates myosin heavy chain. Scale bar, 200 μm.

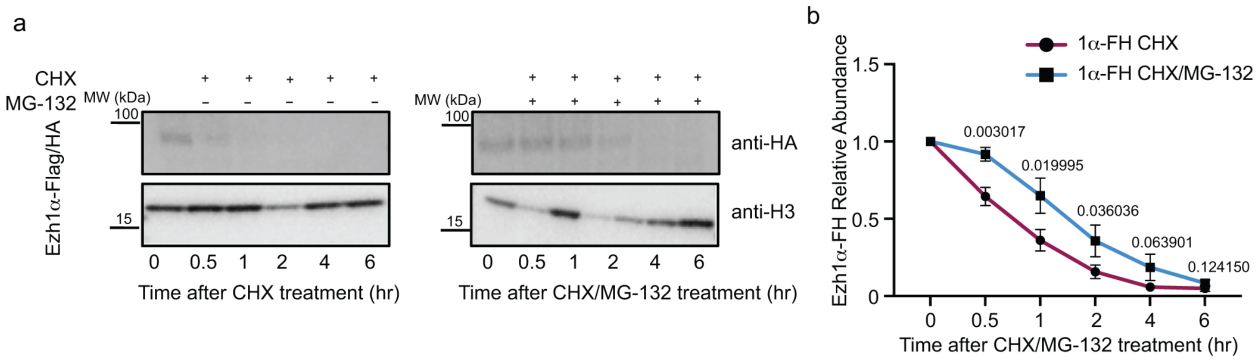

Fig. S8 CHX chasing assay of Ezh1α-FH under normal condition.

a Total proteins were extracted from stable Ezh1α-FH C2C12 cell line at indicated different time points after treatment with 100 μg/ml cycloheximide (CHX) alone or together with 10 μM MG-132. Immunoblot analysis was performed using anti-HA and anti-H3.

b Percentage of remaining Ezh1α-FH level compared with initial protein level was quantified using ImageJ software.

Data shown in (**a**) has been repeated at least three times. Data was expressed in (**b**) as means ± SD from three biological replicates. Values above each bar indicate Student's t-test p value.
