# Supplementary material for: Ubiquitin ligases HUWE1 and NEDD4 cooperatively control signal-dependent PRC2-Ezh1α/β-mediated adaptive stress response pathway in skeletal muscle cells
Source: Epigenetics Chromatin. 2019 Dec 19;12:78. doi: 10.1186/s13072-019-0322-5 (PMC6921592; doi:10.1186/s13072-019-0322-5)
Supplement: Supplementary file 5 — Additional file 5: Table S1. Oligos sequence information used in this study. [file 13072_2019_322_MOESM5_ESM.docx]

| **Primers name** | **Sequence (from 5' to 3')** | | **Purpose** |  |  |
| --- | --- | --- | --- | --- | --- |
| **Primers for constructs and mutagenesis** | | | |  |  |
| Ezh1β-Xho I-POZ-F | | **CTCGAG**  ATGGATATAGCAAGTCCCCCAACTTCCAAATGCATCACA | Ezh1β-FLAG-HA in pOZ-C-FH |  |  |
| Ezh1β-Not I-POZ-R | | **GCGGCCGC**  AGGGGCAGGAGAAAAGAGTCTGGGCACTCCAAGGCCAA |  |  |  |
| Ezh1α-Xho I-POZ-F | | **CTCGAG** ATGAGGAAAATGGATATAGCAAGTCCCCCAACTTCCAAATGCA | Ezh1α-FLAG-HA in pOZ-C-FH |  |  |
| Ezh1α-Not I-POZ-R | | **GCGGCCGC**  GAAGACGTCCGTTTCCCTCTCGATG |  |  |  |
| HA-Ubiquitin-F-Xho I | | **CTCGAG**  atgtacccatacgatgttccggattacgctagcggatccatgcag | HA-Ubiquitin with stop codon in pOZ-C-FH |  |  |
| HA-Ubiquitin-R-Stop-Not I | | **GCGGCCGC**  ctaataaccacctctcagacgcaggaccagg |  |  |  |
| Ezh1β-Xho I-POZ-F | | **CTCGAG**  ATGGATATAGCAAGTCCCCCAACTTCCAAATGCATCACA | Ezh1β-2XT7 with stop codon in pOZ-C-FH |  |  |
| Ezh1β-Not I-2XT7-Stop R | | **GCGGCCGC**  CTAACCCATCTGTTGGCCACCGGTCATAGAAGCCATGCTGGCCGAAGAGCCGCCCATTTGCTGCCCGCCGGTCATAGAAGCCATAGGGGCAGGAGAAAAGAGTCTGGGCACTCCAAGGCCAA |  |  |  |
| Ezh1β-Xho I-POZ-F | | **CTCGAG**  ATGGATATAGCAAGTCCCCCAACTTCCAAATGCATCACA | Ezh1βS560A-2XT7 with stop codon in pOZ-C-FH |  |  |
| Ezh1βS560A-Not I-2XT7-Stop R | | **GCGGCCGC** CTAACCCATCTGTTGGCCACCGGTCATAGAAGCCATGCTGGCCGAAGAGCCGCCCATTTGCTGCCCGCCGGTCATAGAAGCCATAGGGGCAGGAGAAAAGAGTCTGGGCACTCCAAGGCCAACCACTTGGGTAGACGATGG**AGC**AAGCAGGGTGCT |  |  |  |
| Ezh1β-Xho I-POZ-F | | **CTCGAG**  ATGGATATAGCAAGTCCCCCAACTTCCAAATGCATCACA | Ezh1βS560D-2XT7 with stop codon in pOZ-C-FH |  |  |
| Ezh1βS560D-Not I-2XT7-Stop R | | **GCGGCCGC** CTAACCCATCTGTTGGCCACCGGTCATAGAAGCCATGCTGGCCGAAGAGCCGCCCATTTGCTGCCCGCCGGTCATAGAAGCCATAGGGGCAGGAGAAAAGAGTCTGGGCACTCCAAGGCCAACCACTTGGGTAGACGATGG**ATC**AAGCAGGGTGCT |  |  |  |
| Ezh1β-Xho I-POZ-F | | **CTCGAG**  ATGGATATAGCAAGTCCCCCAACTTCCAAATGCATCACA | Ezh1βS560A-FLAG-HA in pOZ-C-FH |  |  |
| Ezh1β-S560A-Not I-R | | **GCGGCCGC**  AGGGGCAGGAGAAAAGAGTCTGGGCACTCCAAGGCCAACCACTTGGGTAGACGATGG**AGC**AAGCAGGGTGCT |  |  |  |
| Ezh1β-Xho I-POZ-F | | **CTCGAG**  ATGGATATAGCAAGTCCCCCAACTTCCAAATGCATCACA | Ezh1βS560D-FLAG-HA in pOZ-C-FH |  |  |
| Ezh1β-S560D-Not I-R | | **GCGGCCGC**  AGGGGCAGGAGAAAAGAGTCTGGGCACTCCAAGGCCAACCACTTGGGTAGACGATGG**ATC**AAGCAGGGTGCT |  |  |  |
| Ezh1α-Xho I-POZ-F | | **CTCGAG** ATGAGGAAAATGGATATAGCAAGTCCCCCAACTTCCAAATGCA | Ezh1α-2XT7 with stop codon in pOZ-C-FH |  |  |
| Ezh1α-2XT7-Stop-Not I-R | | **GCGGCCGC**  CTAACCCATCTGTTGGCCACCGGTCATAGAAGCCATGCTGGCCGAAGAGCCGCCCATTTGCTGCCCGCCGGTCATAGAAGCCATGAAGACGTCCGTTTCCCTCTCGATGCCCACATACTTG |  |  |  |
|  | | | |  |  |
| **Primers for ChIP-qPCR analysis** | | | |  | pGEX-5X-3-JMJ14 (596) |
| mCK enhancer F | TAC TGT TCC ATG TTC CCG GCG AA | | mCK ChIP qPCR |  |  |
| mCK enhancer R | AGG AGC CTA CAG GGT GTG ACT A | |  |  |  |
| MyoG promoter F | TGG CTA TAT TTA TCT CTG GGT TCA | | MyoG promoter ChIP qPCR |  |  |
| MyoG promoter R | GCT CCC GCA GCC CCT CAC ACC | |  |  |  |
| Myh8 F | TAG TGT GTT GGG AAG GGA ATC T | | Myh8 ChIP qPCR |  |  |
| Myh8 R | GCT CCT GTT GGA ACA AAT AAG G | |  |  |  |
| NeuroG1 F | CCT CCC GCG AGC ATA AAT | | NeuroG1 ChIP qPCR |  |  |
| NeuroG1 R | CCT CAG GAC CCC TTA AGT ACG | |  |  |  |
|  |  | |  |  |  |
| **Primers for RT-qPCR analysis** | | | |  |  |
| Gapdh F | AAC ATC AAA TGG GGT GAG GCC | | GAPDH qPCR |  |  |
| Gapdh R | GTT GTC ATG GAT GAC CTT GGC | |  |  |  |
| Myogenin F | AAT GCA CTG GAG TTC GGT C | | MyoG qPCR |  |  |
| Myogenin R | GCA ACA GAC ATA TCC TCC ACC | |  |  |  |
| mCK F | ACT ACA AGC CTC AGG AGG AGT A | | mCK qPCR |  |  |
| mCK R | ATC GCG AAG CTT ATT GTA G | |  |  |  |
| Myh8 F | GAA CTT GAA GGA GAG GTC GA | | Myh8 qPCR |  |  |
| Myh8 R | GAG CAC ATT CTT GCG GTC TT | |  |  |  |
| NEDD4 F | ATG TGG ATG CTG GGA GTT GA | | NEDD4 qPCR |  |  |
| NEDD4 R | CTG CAC GCT GGT AAG GAT TC | |  |  |  |
| HUWE1 F | ACT ACC CCT GTC ACT TCT GC | | HUWE1 qPCR |  |  |
| HUWE1 R | CCT CCT TCA CCA ACC TTT GC | |  |  |  |
| FBXW8 F | CGC CAA GGA GCA CAC ATT AA | | FBXW8 qPCR |  |  |
| FBXW8 R | AGA ACG CAC GTC ACA CAA AA | |  |  |  |
| Atrogin1 F | CAG CAG CCT GAA CTA CGA CG | | Atrogin 1 qPCR |  |  |
| Atrogin1 R | GGC AGT CGA GAA GTC CAG TC | |  |  |  |
|  |  | |  |  |  |
