# Supplementary material for: Ubiquitin ligases HUWE1 and NEDD4 cooperatively control signal-dependent PRC2-Ezh1α/β-mediated adaptive stress response pathway in skeletal muscle cells
Source: Epigenetics Chromatin. 2019 Dec 19;12:78. doi: 10.1186/s13072-019-0322-5 (PMC6921592; doi:10.1186/s13072-019-0322-5)
Supplement: Supplementary file 6 — Additional file 6: Table S2. Primary antibodies information used in this study. [file 13072_2019_322_MOESM6_ESM.docx]

| ***Epitope*** | ***Antibody*** | ***Supplier*** | ***Blocking condition for WB*** | ***Antibody diluition for WB*** |
| --- | --- | --- | --- | --- |
| HUWE1 | PA5-51719 | Thermo Fisher Scientific | 5% milk PBS or TBS Tween 0.1% | 1 : 1.000 |
| CUL7 | PA5-56338 | Thermo Fisher Scientific | 5% milk PBS or TBS Tween 0.1% | 1 : 1.000 |
| NEDD4 | 2740S | Cell Signaling Technology | 5% BSA PBS or TBS Tween 0.2% | 1 : 1.000 |
| FBXW8 | PA5-58565 | Thermo Fisher Scientific | 5% milk PBS or TBS Tween 0.1% | 1 : 1.000 |
| HA | 12158167001 Clone (3F10) | Roche | 5% milk PBS or TBS Tween 0.1% | 1 : 10.000 |
| β actin | SC-47778 (C4) | Santa Cruz | 5% milk PBS or TBS Tween 0.1% | 1 : 10.000 |
| Ezh1β C-term |  | V. Orlando Lab | 5% BSA TBS Tween 0.5% | 1 : 5.000 |
| Ubiquitin | 3933S | Cell Signaling Technology | 5% BSA TBS Tween 0.2% | 1 : 10.000 |
| SUZ12 | 3737 (D39F6) | Cell Signaling Technology | 5% milk TBS Tween 0.1% | 1 : 1.000 |
| EED | 05-1320 (Clone AA 19) | Millipore | 5% milk TBS Tween 0.1% | 1 : 1.000 |
| pSerine | ab9332 | Abcam | 5% milk TBS Tween 0.2% | 1 : 1.000 |
| T7 | 13246 (D9E1X) | Cell Signaling Technology | 5% BSA TBS Tween 0.1% | 1 : 1.000 |
| Histone 3 | ab1791 | Abcam | 5% milk TBS Tween 0.1% | 1 : 10.000 |
| H3K27me3 | 07-449 | Millipore | 5% milk TBS Tween 0.1% | 1 : 10.000 |
| Ezh1α | ab13665 | Abcam | For ChIP assay | For ChIP |
